# Trimester-specific thyroid hormone reference ranges in Sudanese women

**DOI:** 10.1186/s12899-016-0025-0

**Published:** 2016-10-31

**Authors:** Enaam T. Elhaj, Ishag Adam, Mohamed A. Ahmed, Mohamed F. Lutfi

**Affiliations:** 1 Faculty of Applied Medical Science, Gezira University, Wad Madani, Sudan; 2 Faculty of Medicine, University of Khartoum, Khartoum, Sudan; 3 Faculty of Medicine and Health Sciences, Alneelain University, Khartoum, Sudan

**Keywords:** Pregnancy, Reference range, Sudan, Trimester, Thyroid

## Abstract

**Background:**

Trimester-specific reference ranges for T3, T4, and TSH need to be established in different communities. Neither Sudan nor other African countries have established trimester-specific reference ranges for TSH, free T3 (FT3), and free T4 (FT4) in healthy pregnant women. This study aimed to establish trimester-specific reference ranges for TSH, FT3, and FT4 in healthy pregnant Sudanese women.

**Results:**

We performed a longitudinal study, which included 63 women with singleton pregnancies who were followed since early pregnancy until the third trimester. The study was performed in Saad Abu-Alela Hospital, Khartoum, Sudan, during January to October 2014. An equal number of age- and parity-matched non-pregnant women were enrolled as a control group. Basic clinical and obstetrics data were gathered using questionnaires. TSH, FT3, and FT4 levels were measured. Median (5th–95th centile) values of TSH, FT3, and FT4 were 1.164 IU/ml (0.079–2.177 IU/ml), 4.639 nmol/l (3.843–6.562 nmol/l), and 16.86 pmol/l (13.02–31.48 pmol/l) in the first trimester. Median values of TSH, FT3, and FT4 were 1.364 IU/ml (0.540–2.521 IU/ml), 4.347 nmol/l (3.425–5.447 nmol/l), and 13.51 pmol/l (11.04–31.07 pmol/l) in the second trimester. These values were 1.445 IU/ml (0.588–2.460 IU/ml), 4.132 nmol/l (3.176–5.164 nmol/l), and 12.87 pmol/l (9.807–23.78 pmol/l) in the third trimester, respectively. TSH levels increased throughout the trimesters. FT3 and FT4 levels were significantly higher in the first trimester compared with the second and third trimesters. TSH, FT3, and FT4 levels were significantly lower in pregnant women compared with non-pregnant women (*P* < 0.001).

**Conclusions:**

The present study is the first to establish trimester-specific reference ranges of TSH, FT3, and FT4 in Sudanese women with normal pregnancies. Our results suggest that pregnancy is likely to suppress TSH, T3, and T4 levels in healthy women.

## Background

Pregnancy may be associated with alterations in iodine homeostasis and other physiological changes that ultimately result in alteration of thyroid function [1, 2]. Several community-based cohort studies were conducted to establish trimester-specific reference ranges (TSRRs) for T3, T4, and TSH in different communities [3–10]. Studies have shown considerable variations in the thyroid hormone profile among populations of different origin, probably owing to modulatory effects of ethnicity [11], parity [12], body mass index (BMI) [13], iodine insufficiency [1, 2], and certain pregnancy-induced disorders [14, 15] of thyroid function. Establishing TSRRs of TSH, free T3 (FT3), and free T4 (FT4) are therefore crucial for meticulous evaluation of thyroid function during pregnancy. Maternal thyroid function should be maintained within normal limits, especially during the first trimester. In the first trimester, the embryo is completely dependent on the mother’s thyroid hormones for development of the brain [16], as well as other tissues [17, 18].

Many studies have been conducted worldwide to establish TSRRs in different countries [3–10]. However, similar studies in Sudan [19] and other African countries [20] are scarce. A previous study that assessed iodine status in 21 pregnant Sudanese women reported trimester-specific interquartile ranges of TSH, T3, and T4 [19]. However, the sample size was inadequate for establishing TSRRs for thyroid hormones in Sudan. A study in Nigeria evaluated the effect of iodine deficiency on thyroid function during pregnancy, and reported TSRRs of TSH, T3, and T4 based on a small sample size of 27 [20].

To the best of our knowledge, no other studies have been conducted to establish TSRRs for healthy pregnant women either in Sudan or in other African countries. The current study was conducted to establish TSRRs for healthy pregnant Sudanese women. Our findings are expected to assist in accurate interpretation of laboratory results, proper diagnosis, and management of thyroid dysfunction among Sudanese women during pregnancy.

## Methods

We conducted a longitudinal study in Saad Abu-Alela Hospital (Khartoum, Sudan) during January to October 2014. Women with a singleton pregnancy were enrolled early in pregnancy (once their pregnancy was confirmed) and followed until the third trimester. After patients signed an informed consent, the sociodemographic, medical, and obstetrics history (age, parity, gravidity, and gestational age) was recorded from each woman using a questionnaire. Pregnancy and its duration were confirmed by ultrasound, which was conducted by the practicing obstetricians (MAA and IA) as part of antenatal investigations. During the current longitudinal study, an equal number of non-pregnant women (pregnancy was excluded by ultrasound) matched for age and parity were included as controls. Pregnant and non-pregnant women who had a previous history of thyroid disease, hypertension, renal disease, diabetes, liver disease, and those on medication that might affect thyroid function were excluded from the study.

Blood pressure was measured using a sphygmomanometer for the case and control groups. During the first visit (in early pregnancy), maternal weight and height were measured. BMI was calculated and expressed as weight (kg)/height (m^2^). BMI during the first trimester is likely comparable with pre-conception BMI, which was difficult to get in our study population. Hemoglobin was measured for each patient at every visit.

A volume of 5 ml of venous blood was taken from each patient, allowed to clot, centrifuged, and stored at −20 °C until assay of thyroid hormones (TSH, FT3, FT4) using the immunoassay analyzer AIA 360 TOSOH (Japan), guided by the manufacturer’s instructions as described previously [15]. Noteworthy, the aim of present study was to get TSRRs of TSH, FT3 and FT4 regardless of parity or residence. Thus data were not dissected. The reference intervals for TSH, FT3, and FT4 for our laboratory were as follows: TSH, 0.38–4.3 IU/ml; FT3, 3.23–5.13 pmol/l; and FT4, 10.55–20.9 pmol/l.

A total required sample size of 63 women was calculated using a formula for a longitudinal study and the difference in the mean of the proposed variables (TSH, FT3, and FT4 as in our previous study [15]) that would provide 80 % power to detect a 5 % difference at α = 0.05. We assumed that 10 % of women will be lost during follow-up or will not respond.

### Statistical analysis

Data were entered into a computer using SPSS for Windows (version 16.0). The 5th–95th centile and median were calculated for levels of TSH, FT3, and FT4. Levels of TSH, FT3, and FT4 were not normally distributed (assessed using the Shapiro–Wilk normality test), and differences were assessed using the Kruskal–Wallis H test (Mann–Whitney U test between two groups). A *P* value < 0.05 was considered significant.

## Results

Among 75 pregnant women who were initially enrolled, 63 (84.0 %) completed the follow-up until the third trimester. The rest (16.0 %) of the patients were lost during follow-up because of a change in address.

Approximately half of these women were primiparous (*n* = 34.0, 54.0 %), the majority were housewives (*n* = 45, 71.4 %), and few of them had a rural residence (*n* = 9, 14.3 %). Hemoglobin level and the other basic characteristics of the women are shown in Table 1.

**Table 1:** Mean (SD) of the characteristics of the enrolled pregnant women (*n* = 63)

Variable	Mean (SD)
Age, years	27.0 (4.9)
Parity	0.8 (1.1)
Gravidity	2.5 (4.0)
Body mass index	27.9 (5.4)
Gestational age, weeks	
First trimester	10.8 (2.9)
Second trimester	21.5 (4.0)
Third trimester	31.2 (3.4)
Hemoglobin, gm/dl	
First trimester	10.6 (1.0)
Second trimester	10.6 (0.6)
Third trimester	10.9 (1.1)

TSH, FT3, and FT4 levels were significantly lower in pregnant women compared with non-pregnant women (Table 2). Median (5th–95th centile) values of TSH, FT3, and FT4 in pregnant women were 1.164 IU/ml (0.079–2.177 IU/ml), 4.639 nmol/l (3.843–6.562 nmol/l), and 16.86 pmol/l (13.02–31.48 pmol/l) in the first trimester. Median values of TSH, FT3, and FT4 were 1.364 IU/ml (0.540–2.521 IU/ml), 4.347 nmol/l (3.425–5.447 nmol/l), and 13.51 pmol/l (11.04–31.07 pmol/l) in the second trimester. These values were 1.445 IU/ml (0.588–2.460 IU/ml), 4.132 nmol/l (3.176–5.164 nmol/l), and 12.87 pmol/l (9.807–23.78 pmol/l) in the third trimester, respectively.

**Table 2:** Median (5th–95th centile) values of thyroid hormones in pregnant and non-pregnant Sudanese women

Variable	Non- pregnant	Trimester-I	Trimester-II	Trimester-III	*P*
		Mean = 10.8 weeks	Mean =21.5 weeks	Mean =31.2 weeks	
TSH, IU/ml	2.080 (0.776–4.550)	1.164 (0.079–2.177)	1.364 (0.540–2.521)	1.445 (0.588–2.460)	Non- Pregnant vs. Pregnant *P* < 0.001
					Trimester-I vs. Trimester-II P = 0.042
					Trimester-I vs. Trimester-III P = 0.007
					Trimester-II vs. Trimester-III P = 0.482
Free T3, pmol/l	5.176 (4.156–6.095)	4.639 (3.843–6.562)	4.347 (3.425–5.447)	4.132 (3.176–5.164)	Non- Pregnant vs. Pregnant *P* < 0.001
					Trimester-I vs. Trimester-II *P* < 0.001
					Trimester-I vs. Trimester-III *P* < 0.001
					Trimester-I vs. Trimester-III P = 0.064
Free T4, pmol/l	16.34 (12.6–19.9)	16.86 (13.02–31.48)	13.51 (11.04–31.07)	12.87 (9.807–23.78)	Non- Pregnant vs. Pregnant *P* < 0.001
					Trimester-I vs. Trimester-II P = 0.008
					Trimester-I vs. Trimester-III *P* < 0.001
					Trimester-I vs. Trimester-III P =0.078

**Fig. 1:**
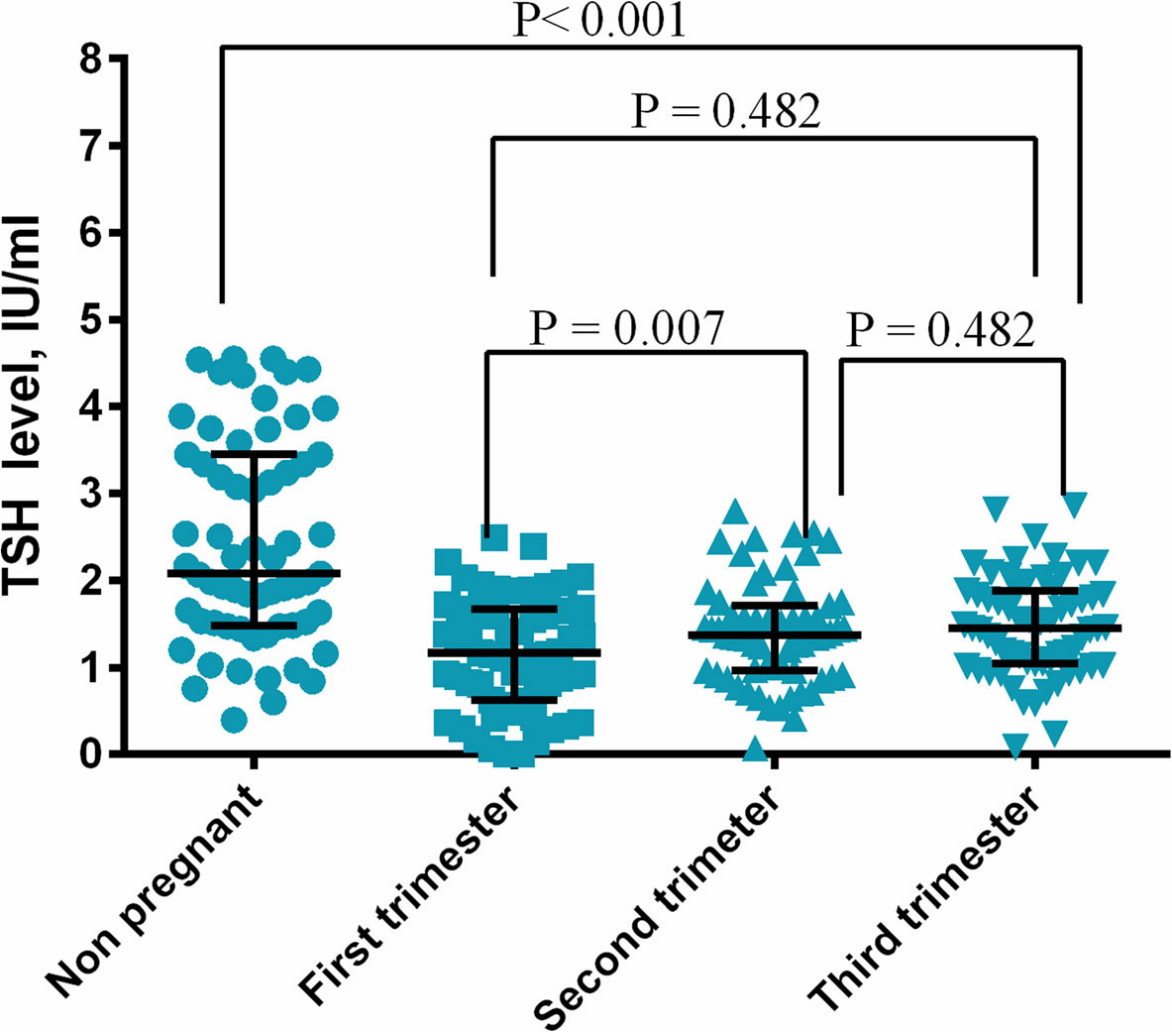
Distribution of TSH levels among controls and different trimesters in pregnant women

TSH levels increased over the trimesters, with significantly higher TSH levels in the second and third trimesters compared with the first trimester (Table 2, Fig. 1).

**Fig. 2:**
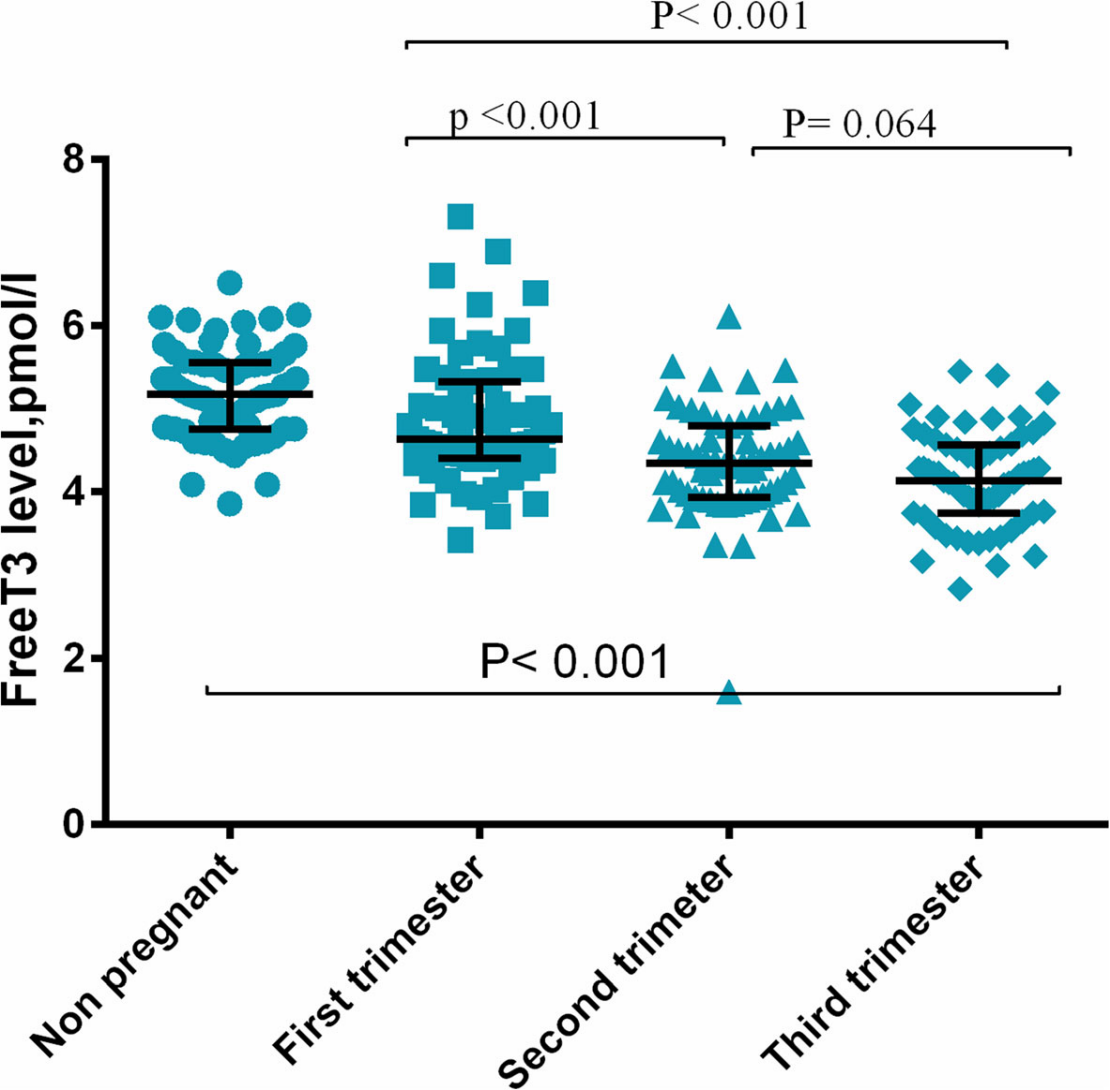
Distribution of T3 levels among controls and different trimesters in pregnant women

FT3 levels were significantly higher in the first trimester compared with the second and third trimesters. However, no significant difference was observed in FT3 levels between the second and third trimesters (Table 2 and Fig. 2).

**Fig. 3:**
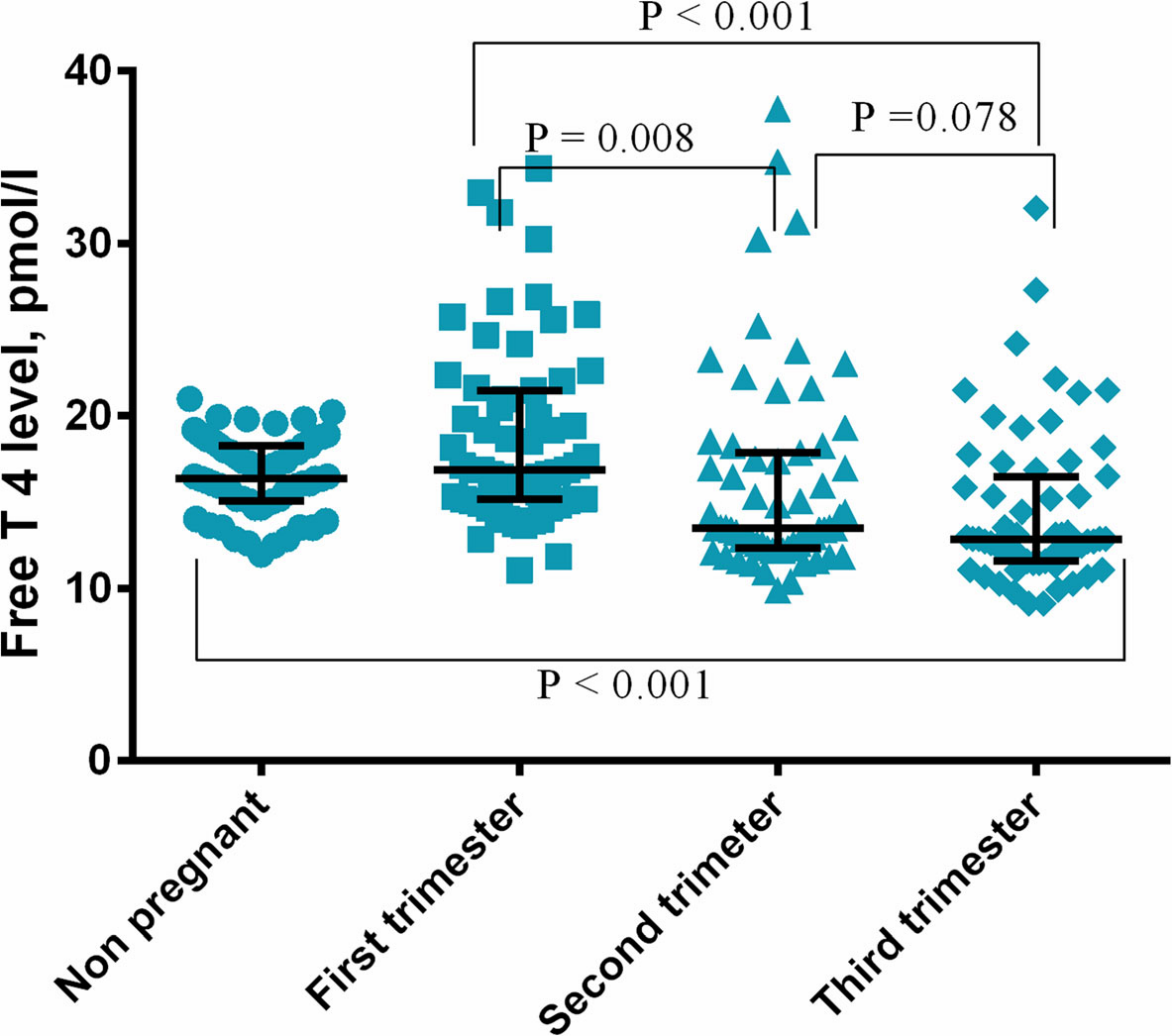
Distribution of T4 among controls and different trimesters in pregnant women

FT4 levels showed significant variation between the first and second trimesters, as well as between the first and third trimesters. FT4 levels were lowest during the third trimester. No significant difference in FT4 levels was observed between the second and third trimesters (Table 2 and Fig. 3).

## Discussion

The present study is the first to establish TSRRs of TSH, FT3, and FT4 in healthy Sudanese pregnant women. A previous study by Eltom et al. assessed iodine status during pregnancy in Sudan [19]. They observed comparable trimester-specific TSH levels, but lower T3 and T4 interquartile ranges, compared with our results (Table 3). They evaluated 21 pregnant Sudanese women, which was an inadequate sample size to establish TSRRs for thyroid hormones in Sudan. Similar to the present findings, they found that TSRRs of T3 and T4 were lowest in the third trimester. In contrast, maximum TSH levels were reported in the second trimester [19]. To the best of our knowledge, there have been no studies on TSRRs for healthy pregnant women in any of the African countries. A previous Nigerian study examined the effect of iodine deficiency on thyroid function during pregnancy [20]. TSRRs of TSH, T3, and T4 were calculated after evaluating 27 African women with a normal pregnancy [20]. As shown in Table 4, TSRRs of TSH in all trimesters were higher in the Nigerian study compared with the current study on pregnant Sudanese women. In contrast, thyroid hormone levels were higher in pregnant Sudanese women, especially T4, compared with Nigerian women. TSRRs of TSH, FT3, and FT4 levels in pregnant Sudanese women showed a unique trend compared with previous studies in other countries. TSRRs of TSH in Sudanese are comparable with some Western countries (Table 4) [3, 4], but relatively lower compared with some Arab [5], Persian [6], and Eastern countries [7, 8]. TSRRs of T4, and to some extent T3, are higher in Sudanese women compared with other countries (Table 4) [3–10].

**Table 3:** Comparison of interquartile ranges of thyroid hormones between our study and a previous Sudanese report

		Trimester I	Trimester II	Trimester III
Present study	TSH, IU/ml	0.6–1.6	0.9–1.7	1.0–1.8
	T3, nmol/L	4.4–5.3	3.9–4.7	3.7–4.5
	T4, pmol/L	15.1–21.4	12.3–17.8	11.5–16.4
Eltom et al. [19].	TSH, IU/ml	0.5–1.5	0.7–1.8	0.6–1.6
	T3, nmol/L	2.1–3.2	2.2–3.5	2.2–2.9
	T4, pmol/L	9.6–13.4	8.6–10.8	9.4–12.6

**Table 4:** Comparison of thyroid hormones between our study from Sudan and previous reports from other countries

	Percentile used	TFT	Non-pregnant	Trimester I	Trimester II	Trimester III
Sudan	5–95	TSH, IU/ml	0.78–4.55	0.08–2.18	0.54–2.52	0.59–2.46
		T3, nmol/L	4.16–6.10	3.84–6.56	3.43–5.45	3.18–5.16
		T4, pmol/L	12.6–20.0	13.00–31.53	11.07–31.2	9.78–23.81
Nigeria [20]	5–95	TSH, IU/ml	–	0.5–5.0	0.5–5.0	0.5–5.0
		T3, nmol/L	–	1.7–3.5	1.86–3.54	1.9–3.94
		T4, pmol/L	–	6.1–19.7	8.5–23.3	8.9–23.3
UAE [5]	2.5–97.5	TSH, IU/ml	0.21–6.9	0.17–5.9	0.04–9.3	0.3–4.32
		T3, nmol/L	–	–	–	–
		T4, pmol/L	8–18	8.4–19.3	8.9–24.6	9.8–18.6
Iran [6]	5–95	TSH, IU/ml	–	0.2–3.9	0.5–4.1	0.6–4.1
		T3, nmol/L	–	–	–	–
		T4, pmol/L	–	8.5–19	9.7–21	8.7–20.4.
Turkey [9]	2.5–97.5	TSH, IU/ml	0.47–4.70	0.49–2.33	0.51–3.44	0.58–4.31
		T3, nmol/L	2.96–5.48	3.80–5.81	3.69–5.90	3.67–5.81
		T4, pmol/L	10.68–18.23	10.30–18.11	10.30–18.15	10.30–17.89
Switzerland [3]	2.5–97.5	TSH, IU/ml	0.35–4.94	0.06–2.83	0.20–2.79	0.31–2.90
		T3, nmol/L	–	3.52–6.22	3.41–5.78	3.33–5.59
		T4, pmol/L	9–19.1	10.5–18.3	9.5–15.7	8.6–13.6
Spain [10]	2.5–97.5	TSH, IU/ml	–	0.10–2.65	0.12–2.64	0.23–3.56
		T3, nmol/L	–	3.44–6.80	3.79–6.42	3.46–6.39
		T4, pmol/L	–	10.61–17.76	9.1–14.67	8.49–15.6
India [7]	5–95	TSH, IU/ml	–	0.6–5.0	0.43–5.78	0.74–5.7
		T3, nmol/L	–	1.92–5.86	3.2–5.7	3.3–5.18
		T4, pmol/L	–	12.0–19.45	9.48–19.58	11.3–17.71
China [8]	2.5–97.5	TSH, IU/ml	0.54–4.20	0.16–3.78	0.34–3.51	0.34–4.32
		T3, nmol/L	–	2.9–5.0	2.9–4.6	2.9–4.5
		T4, pmol/L	9.2–16.9	10.9–17.7	9.3–15.2	7.9–14.1
USA [4]	2.5–97.5	TSH, IU/ml	–	0.24–2.99	0.46–2.95	0.43–2.78
		T3, nmol/L	–	–	–	–
		T4, pmol/L	–	–	–	–

TSH and thyroid hormones show day/night [21] and seasonal variation [22, 23]. These hormones are also affected by iodine intake [23] and excretion [24], ambient temperature [25], luminosity and other ecological/genetic factors [26–28]. These biological and environmental factors are unlikely to be comparable worldwide, which could explain the variation in TSRRs of TSH, T3, and T4 in different countries. The variation in TSRRs can also be explained by variable accuracy of the techniques used for thyroid hormonal assays [29, 30] and a lack of standardized preanalytical conditions (i.e., procedure and timing of blood sampling) [31, 32]. Ideally, blood samples for thyroid hormone assays should be collected without the aid of a tourniquet from a rested individual in the fasting state [30]. Blood samples should be taken at the same hour of the day to avoid the effect of circadian rhythm on TSH, T3, and T4 [21].

Table 4 shows that levels of TSH, FT3, and FT4 were lower in pregnant women compared with the control group. In our study, a maximum decrease in thyroid function was achieved in the second trimester, with no significant change in thyroid hormone levels up to the end of pregnancy. In a Turkish study, TSH levels of pregnant women were significantly lower than those of non-pregnant controls in the first trimester [9] (Table 4). However, TSH levels were comparable with non-pregnant levels in the second trimester, and higher than non-pregnant levels in the third trimester. This Turkish study also showed that FT3 and FT4 levels were comparable in the three trimesters [9]. Additionally, T3 levels were higher throughout pregnancy, but T4 levels were lower in the second and third trimesters compared with non-pregnant controls. Chinese pregnant women showed a similar pattern of trimester-specific TSH changes that were observed in Turkish women, but FT4 and FT3 levels decreased as pregnancy progressed [8] (Table 4). A previous study that examined pregnant women with different ethnicities in the United Arab Emirates showed that lower TSH levels in the first trimester increased to non-pregnant control levels in the third trimester [5]. Additionally, FT4 showed a steady decrease throughout pregnancy (Table 4). The findings of the United Arab Emirates study were reproduced in another two studies that intended to establish TSRRs of thyroid hormones in Korean [33] and Irish [34] women.

The physiological basis of the pattern of changes in TSH, FT3, and FT4 during pregnancy has been extensively studied towards the end of the last century [35, 36]. Most previous studies attributed variations in the levels of thyroid hormones throughout pregnancy to non-pituitary hormonal control mechanisms [37]. Researchers have hypothesized that human chorionic gonadotropin (hCG) hormone can stimulate TSH receptors, with subsequent release of T3 and T4 from follicular thyroid cells during the first trimester [38]. Thyroid hormones negatively feedback on the pituitary gland and thus decrease TSH levels [39]. High estrogen levels induced by hCG may markedly decrease free T3 and T4 levels by increasing their transport proteins. As a result, TSH is expected to increase, contradicting the thyrotropic action of hCG [40]. These contradictory actions of hCG on TSH levels suggest that the hypothesis that attributes the second and third trimester TSH surge to hCG withdrawal is questionable [41, 42]. The current study and at least two other studies showed a significant decrease in TSH levels in the second trimester, but were not able to explain this finding [43, 44]. Notably, a concurrent decrease in TSH levels with FT3 and FT4 levels excludes iodine insufficiency as a probable cause of decreased thyroid hormones in the Sudanese pregnant women in our study. This is because, in such conditions, TSH is expected to increase as a compensatory mechanism for decreased T3 and T4 levels. To the best of our knowledge, a further decrease in TSH levels in the second trimester is unclear and may preclude hCG withdrawal as a probable explanation for low hCG levels in the first trimester.

Notably, TSRRs that were established in the present study were derived by following up Sudanese women with a normal pregnancy throughout the three trimesters. Our study design avoids between-individual variation as a possible confounder for the apparent differences in TSRRs of TSH, T3, and T4 demonstrated in this study. However, within-individual variation (e.g., seasonal variation) [22, 23] of TSH and thyroid hormones remains a possible bias for the current findings. Another limitation of this study is that we did not evaluate iodine homeostasis in pregnant women. However, a simultaneous decrease in TSH and thyroid hormones exclude iodine insufficiency as a probable explanation of hypothyroid function in pregnant women in our study. This is because in such conditions, decreased T3 and T4 levels are expected to feedback positively on TSH and increase its level. Evaluation of urine iodine concentrations in future studies may provide further verification of this hypothesis.

## Conclusions

The present study is the first to establish TSRRs of TSH, FT3, and FT4 in Sudan. Pregnancy is likely to suppress TSH, T3, and T4 levels in Sudanese women. TSRRs of TSH in Sudan are comparable with some Western countries, but are relatively lower compared with some Arab, Persian, and Eastern countries. However, TSRRs of thyroid hormones are higher in Sudan compared with other countries. Further research that also considers iodine homeostasis in pregnant women may provide further support for the conclusions of the present study.

## Abbreviations

BMI: Body mass index
FT3: Free triiodothyronine
FT4: Free tetraiodothyronine
hCG: Human chorionic gonadotropin
T3: Triiodothyronine
T4: Tetraiodothyronine
TSH: Thyroid-stimulating hormone
TSRRs: Trimester-specific reference ranges
